# Developing a clinically relevant classification to predict mortality in severe leptospirosis

**DOI:** 10.4103/0974-2700.66519

**Published:** 2010

**Authors:** Senaka Rajapakse, Chaturaka Rodrigo, Rashan Haniffa

**Affiliations:** Department of Clinical Medicine, Faculty of Medicine, University of Colombo, Colombo, Sri Lanka; 1 University Medical Unit, National Hospital of Sri Lanka, Colombo, Sri Lanka; 2 University College London, London, UK

**Keywords:** Leptospirosis, mortality, severity

## Abstract

**Background:**

Severe leptospirosis requires critical care and has a high mortality. We reviewed the literature to identify factors predicting mortality, and such predictors were classified according to the predisposition, infection, response, organ dysfunction (PIRO) concept, which is a risk stratification model used in severe sepsis.

**Material and Methods:**

PUBMED was searched for all articles (English), with the key word leptospirosis in any field, within the last 20 years. Data were collected from 45 relevant papers and grouped into each component of the PIRO model.

**Results:**

The following correlated with increased mortality: predisposition – increasing age and chronic alcoholism; infection - leptospiraemic burden; response - hemodynamic disturbances, leukocytosis; organ dysfunction – multiple organ dysfunction syndrome, pulmonary involvement and acute renal failure.

**Conclusions:**

Further research is needed to identify the role of infecting serovars, clinical signs, inflammatory markers, cytokines and evidence of hepatic dysfunction as prognostic indicators. It is hoped that this paper will be an initiative to create a staging system for severity of leptospirosis based on the PIRO model with an added component for treatment-related predictors.

## BACKGROUND

Leptospirosis is a zoonotic spirochaetal illness with considerable morbidity and mortality in areas of high prevalence. The causative organism is classified in the genus *Leptospira*. Initially, this genus was thought to have only two species. Currently, nine pathogenic species of Leptospira are identified, with another five being classified as “intermediate,” bringing the total number of possible pathogenic species to 14.[1] Rodents, marsupials or any mammal have the potential to be the animal reservoir. The organisms are excreted to the environment via the urine of the host. Transmission to humans can be direct (inoculation via tissues, body fluids and urine of infected animals) or indirect (entry via mucosal surfaces or breached skin from sources contaminated with the causative agent).[2]

An accurate assessment of the disease burden of leptospirosis is not easy due to diagnostic difficulties and under-reporting. The estimated incidence differs as to whether an endemic or a non-endemic area is considered (it is endemic in areas with high rainfall, close human contact with livestock, poor sanitation with workplace exposure to the organism).[3] Such localities, which include pockets in Central America, the Indian subcontinent, Oceania and the Caribbean, have an estimated annual incidence of around 25 clinical infections per 100,000 of population (in contrast to 1 per 100,000 per year in non-endemic areas).[3]

Many infections are asymptomatic or pass off as a mild febrile episode. However, a small proportion develops a severe form of the disease with multiple organ dysfunction. The case fatality ratio in severe disease can exceed 40%.[4] Weil's disease, a severe form of leptospirosis, is characterized by jaundice, hemorrhage and renal failure. In addition, the central nervous system and the cardiovascular system are also affected.[4]

The objectives of this review are two-fold. Firstly, it summarizes the mortality predictors for leptospirosis published by different authors. While some indicators are repeatedly cited as predictors of mortality, the significance of others is questionable. Secondly, an attempt is made to classify such predictors into a clinically relevant classification (based on the predisposition, infection, response, organ dysfunction [PIRO] model).

## PREDICTORS OF MORTALITY ;THE IMPORTANCE OF CLASSIFICATION AND THE PIRO CONCEPT

It is our opinion that a simple detailed description of mortality indicators will not be of much use to a clinician in his practice. The clinician will be more interested in a systematic manner of application of such knowledge to predict mortality and identify the “at risk” patients early. The ideal method to integrate such information to an applicable clinical tool is to develop a valid classification and a scoring system.

The PIRO model is a risk stratification system for severe sepsis. PIRO stands for predisposition (P), infection (I), response (R) and organ dysfunction (O)[5] (Similar to the TNM [tumor, nodes, metastases] classification for staging of malignancy). Severe leptospirosis results in sepsis with multiple organ dysfunction syndrome (MODS); however, the pathogenesis and clinical manifestations of leptospirosis are distinct from other bacterial infections resulting in severe sepsis and other viral infections such as dengue that result in widespread systemic manifestations; leptospirosis causes very specific organ damage to the liver, kidneys, central nervous system (CNS) and heart; predictors of survival that are applicable to these diseases are hence likely to be different in the case of leptospirosis infection. We sought to develop a classification system based on the PIRO concept, which is specifically applicable and clinically relevant in predicting the severity of leptospirosis.

The PIRO staging system for severe sepsis not only classifies prognostic indicators but also provides a scoring system to quantify the probable outcome (each category is assigned a staging number, i.e., P1, P2, R1, etc., with higher numbers predicting increased mortality). This review classifies the prognostic indicators for severe leptospirosis according to the PIRO model but stops short of developing a scoring system. Available data were inadequate, and non-comparable due to differences in study methodology, to develop a valid scoring system. It is our opinion that such quantification is best handled by a well-designed, dedicated, prospective study rather than by a literature review. Still, this work serves as a concept paper to show the possibility and relevance of such a scoring system.

## MATERIAL AND METHODS

A PUBMED search was performed for all articles with the key word “leptospirosis” in any field. The search was restricted to articles published in English within the last 20 years (1989–May 2009) as they would contain more recent data. There were 1898 abstracts in the original search with these restrictions. The software, Endnote X1, was used to filter articles. Bibliographies of cited literature were also searched. All abstracts were read through independently by the three authors and relevant papers were identified for review of the full papers. Related papers were also included. We reviewed 163 selected papers.

These sources were screened for a well-described methodology, accurate statistical analysis and an adequate sample size where relevant. Identification of prognostic indicators was performed by three reviewers independently blinded to each other. Later, they were classified into each component of the PIRO model by consensus. While such a classification was possible, the development of a scoring system based on it was not possible for the reasons mentioned above (non-comparability of data). Data sources included reviews published in core clinical journals, cohort studies, interventional studies, case control studies, cross-sectional analysis and epidemiological data. Suitable data were available in 45 papers from the initially selected 163. A summary of the cited literature is shown in Tables 1–4.

**Table 1:** Summary of studies for predisposition

Predisposition				
Author	Study design, locality and sample size	Year	Mortalities and case fatality rates (%)	Prognostic factor positively linked with mortality
Cetin **et al**.[6]	*n* = 16, retrospective analysis, Turkey	2004	4, 25	Old age
Chawla *et al*.[7]	*n* = 60, prospective study, India	2004	31, 52	Male sex, age over 50, alcohol dependence
Garcia *et al*.[13]	*n* = 26, cohort study, Spain	2000	3, 11.5	Cigarette smoking
Jansen *et al*.[10]	*n* = 338, retrospective analysis, Germany	2005	14, 4.1	Sex is not a mortality indicator
Ko *et al*.[8]	*n* = 326, prospective study, Brazil	1999	50, 15	Age over 37 years
Lopes *et al*.[9]	*n* = 840, retrospective study, Brazil	2004	121, 14.4	Mortality is higher for adults than pediatric patients and adolescents and among adults with increasing age
Papa *et al*.[11]	*n* = 123, retrospective study, Greece	2009	10, 8.1	Gender is not associated with mortality
Pertuiset *et al*.[12]	*n* = 249, prospective study, La Reunion Islands	1988		Chronic alcoholism
Spichler *et al*.[4]	*n* = 370, retrospective case control study, Brazil	2008	89, 24.1	Old age

**Table 2:** Summary of studies for infection

Infection				
Author	Study design, locality and sample size	Year	Mortalities and case fatality rates	Prognostic factor positively linked with mortality
Truccolo *et al*.[19]	Prospective study	2001		Leptospiraemia >10^4^ leptospires/ml
Segura *et al*.[20]	Prospective study in urban and rural areas of Peru, *n* = 321	2005		Leptospiraemia >10^4^ leptospires/ml

**Table 3:** Summary of studies for response

Response				
Author	Study design, locality and sample size	Year	Mortalities and case fatality rates (%)	Prognostic factor positively linked with mortality
Doudier *et al*.[22]	*n* = 71, retrospective study, France	2006	5, 7	Hypotension
Dupont *et al*.[25]	*n* = 68, retrospective analysis, French West Indies	1997	12, 18	White blood cell count >12,000
Marotto *et al*.[24]	*n* = 42, prospective cohort study, Brazil	1999	23, 55	Hemodynamic disturbance
Mathew *et al*.[27]	*n* = 31, prospective and retrospective study, India	2005	8, 25.8	Raised cerebrospinal fluid protein
Panaphut *et al*.[23]	*n* = 121, prospective cohort study, Thailand	2002	17, 14.1	Hypotension
Pappachan *et al*.[21]	*n* = 282, prospective study, India	2004	17, 6.03	Muscle tenderness, tachycardia
Pertuiset *et al*.[12]	*n* = 249, prospective study, La Reunion Islands	1988		Abdominal pain, respiratory rate >24
Spichler *et al*.[4]	*n* = 370, retrospective case control study, Brazil	2008	89, 24.1	Platelet count >70,000
Tajiki *et al*.[26]	*n* = 18, case control study, Brazil	1996	4, 22	Increased tumour necrosis factor α

**Table 4:** Summary of studies for organ dysfunction

Organ dysfunction				
Author	Study design, locality and sample size	Year	Mortalities and case fatality rates (%)	Prognostic factor positively linked with mortality
Bharadwaj *et al*.[33]	*n* = 152, prospective study, India	2002	11, 7.2	Pulmonary and renal involvement
Budiono *et al*.[31]	*n* = 55, retrospective cohort study, Indonesia	2009	8, 14.5	Pulmonary involvement, meningismus
Cetin *et al*.[6]	*n* = 16, retrospective analysis, Turkey	2004	4, 25	High serum bilirubin (>36 mg/dl), high serum potassium, oliguria
Chang *et al*.[28]	*n* = 11, prospective study, Taiwan	2005	5, 45.5	Aspartate transaminase/alanine transaminase ratio is high in late disease
Chawla *et al*.[7]	*n* = 60, prospective study, India	2004	31, 52	MODS*, ARDS†, acidosis on blood gas
Clerk *et al*.[50]	*n* = 38, prospective study, India	2002	6, 15.8	Pulmonary involvement
Covic *et al*.[45]	*n* = 58, retrospective study, Moldova	2003	15, 25.9	MODS*, altered level of consciousness, bleeding diathesis
Daher *et al*.[36]	*n* = 110, retrospective analysis, Brazil	1999	24, 22	Oliguria
Doudier *et al*.[22]	*n* = 71, retrospective analysis, France	2006	5, 7	Oliguria, abnormal chest auscultation
Dupont *et al*.[25]	*n* = 68, retrospective analysis, French West Indies	1997	12, 18	Repolarization abnormalities on electrocardiogram, alveolar infiltrates on chest X-ray, dyspnoea, oliguria
Esen *et al*.[39]	*n* = 72, retrospective analysis, Turkey	2004	12, 16.7	Altered mental status, serum potassium levels at admission
Ko *et al*.[8]	*n* = 326, prospective study, Brazil	1999	50, 15	Altered mental status, renal and respiratory insufficiency
Lopes *et al*.[9]	*n* = 442, retrospective study, Brazil	2001	64, 14.5	Serum K at admission
Marotto *et al*.[24]	*n* = 42, prospective cohort study (only patients with acute lung injury), Brazil	1999	23, 55	Serum creatinine >265.2 µmol/l, serum K >4.0 meq/l
Mathew *et al*.[27]	*n* = 31, prospective and retrospective study, India	2005	8, 25.8	Deep altered sensorium at presentation
Paganin *et al*.[32]	*n* = 147, retrospective study, La Reunion Islands	2006	19, 6.1	Need for mechanical ventilation
Panaphut *et al*.[23]	*n* = 121, prospective cohort study, Thailand	2002	17, 14.1	Hyperkalaemia, oliguria, presence of pulmonary rales
Papa *et al*.[11]	*n* = 123, retrospective study, Greece	2009	10, 8.1	Pulmonary involvement
Pappachan*et al*.[21]	*n* = 282, prospective study, India	2004	17, 6.03	Hyperkalaemia, meningism, serum Bilirubin >15 mg/dl, disorientation
Pertuiset *et al*.[12]	*n* = 249, prospective study, La Reunion Islands	1988		Radiographic lung disease, clinical or electrocardiographic markers of heart involvement, kidney failure
Shenoy *et al*.[30]	*n* = 30, prospective study, India	2009	11, 36.6	Pulmonary quadrant involvement, MODS*
Spichler *et al*.[4]	*n* = 370, retrospective case control study, Brazil	2008	89, 24.1	Creatinine >3 mg/dl, pulmonary involvement, oliguria
Vieira *et al*.[40]	*n* = 35, prospective study (ICU sample with ARF), Brazil	2002	18, 51	MODS*, bicarbonate level

* MODS, multiorgan dysfunction syndrome

† ARDS, acute respiratory distress syndrome

## COMPREHENSIVE DISCUSSION OF MORTALITY PREDICTORS WITH CONCURRENT CLASSIFICATION INTO THE PIRO MODEL

The following four sections are titled according to the four components of the PIRO model. Mortality predictors for leptospirosis reported by various authors are discussed under each subtopic.

### Predisposition

Three mortality indicators were reported in the literature that can be classified under predisposition: age, alcohol dependence and male sex. A summary of the studies in this regard is given in table 1.

Five studies have reported age as an indicator. Cetin *et al*.^6^ published a retrospective analysis of 16 patients with leptospirosis and renal failure. Survivors had a mean age of 34 years, while the deceased had a mean age of 60.7 years (Mann Whitney U-test, 0.008). Chawla *et al*.[7] described age over 50 years as a mortality indicator in a sample of 60 critically ill patients in an intensive care unit (ICU) setting. Ko *et al*.0,[8] in a prospective study of 396 clinically defined cases, suggest that age over 36 years is significantly associated with death (OR, 4.25). Lopez *et al*.,[9] in a retrospective study of 840 leptospirosis patients, concluded that the odds of death adjusted for gender, jaundice, duration of symptoms, serum urea and serum creatinine were almost four times higher for the adult group than for the pediatric group (OR, 3.94). Among adults also, increasing age was significantly and independently associated with death. Spichler *et al*.[4] showed that age over 40 is a risk factor for death (retrospective case control study with 370 patients).

Only Chawla *et al*.[7] reported male sex as an independent predictor of death. Data from two other studies[10,11] failed to find such an association.

Chronic alcoholism was reported to be significantly associated with death in two studies.[7,12] Martinez *et al*.,[13] in a retrospective analysis of 17 patients with pulmonary involvement, found cigarette smoking to be significantly associated with disease severity but not mortality.

On analyzing the available evidence, the strongest predictor of mortality under “predisposition” is increasing age. The evidence for poor outcome among males is limited (two large retrospective studies failed to show such an association).[10,11] Jansen *et al*.[10] suggested that male sex is significantly associated with severity of disease but does not predict mortality. Two large prospective studies showed that chronic alcoholism is an important predictor of mortality, although most other studies do not mention this in the published literature. Whether this increased risk is due to leptospirosis *per se* or due to hepatic dysfunction on a background of alcoholic liver disease is not clear.

While there are many predisposing factors for contracting leptospirosis (walking barefoot, contact with farm animals and rodents, wet soil around the house, activities in forests, rainfall), none of these have been reported to predict outcome after acquiring the infection.[14–18] Male sex and age have been cited as predictors of infection as well as mortality.

### Infection

The classifiable material under this topic is minimal [Table 2]. Trucculo *et al*.[19] measured the number of leptospires (by polymerase chain reaction) recovered from the blood of patients with leptospirosis, and showed that >10^4^ leptospires/ml were associated with death. These findings were confirmed by Segura *et al*.[20] in an independent, prospective population-based study.

### Response

We encountered difficulty in differentiating mortality indicators under response and organ dysfunction categories as such a division seemed arbitrary. Therefore, the items categorized under the response section are restricted to components of severe inflammatory response syndrome (SIRS) and a few other indicators such as abdominal pain and muscle tenderness [Table 3].

Pertuiset *et al*.[12] report tachypnoea (respiratory rate over 24/min) as a predictor of mortality while Pappachan *et al*.[21] identified tachycardia as a risk indicator (OR, 4.1). Hypotension was associated with mortality in three studies.[22–24] Leukocytosis (total count above 12,000) has also been reported as a mortality predictor by some.[25]

Abdominal pain is also reported as a mortality indicator by Pertuiset *et al*.,[12] although other studies do not show supportive evidence. Pappachan *et al*.[21] describe generalized muscle tenderness as a mortality predictor (*P* = 0.03).

Other indicators classifiable under this section include high tumor necrosis factor alpha (TNF α) levels (Tajiki *et al*., *P* = 0.02), platelet count <70,000 (Spichler *et al*., OR: 2.6) and high cerebrospinal fluid protein levels in patients with neuroleptospirosis (Mathew *et al*., *P* < 0.001).[4,26–28]

Of all these indicators, only hypotension was confirmed as a predictor of mortality in three independent studies with an adequate sample size.[22–24] Other associations such as tachycardia, leucocytosis and TNF α levels have not been confirmed by independent studies. However, Niwatthayakul *et al*.[29] observed that in a series of 34 patients with renal failure, TNF α levels were high in severely ill patients. This is an area for further research. It is notable that markers of inflammation such as C-reactive protein and erythrocyte sedimentation rate have not been adequately assessed with regard to mortality.

### Organ dysfunction

Three organ systems are commonly cited as involved in severe leptospirosis: the lungs, the kidneys and the CNS [Table 4].

Although relatively uncommon, pulmonary involvement has been repeatedly and consistently incriminated as a mortality predictor in more than 10 studies. Pertuiset *et al*.[12] and Dupont *et al*.[25] reported that radiographical evidence of pulmonary involvement is a predictor of mortality. Similarly, Shenoy *et al*.,[30] in a prospective study, reported that mortality correlates positively with the number of lung quadrants involved on the chest radiograph. Abnormal chest auscultation,[22] dyspnoea[23] and the presence of pulmonary rales[25] are also associated with increased mortality. Several other studies showing an association between pulmonary involvement and mortality are summarized in Table 4.[4,11,31–33]

While many manifestations of lung involvement are mild, pulmonary hemorrhage is associated with a high mortality. Patients also die from acute lung injury (ALI), acute respiratory distress syndrome and combined MODS. Respiratory symptoms appear on the 4^th^–6^th^ days of disease and can progress rapidly to death within 72 h. Mortality rates from different studies vary between 30% and 60%. Different aspects of lung involvement such as clinical signs, radiographical evidence and ALI are all predictors of mortality.[34,35]

Acute renal failure (ARF) and its manifestations are also reported as mortality indicators. Oliguria and anuria are cited as independent predictors of mortality in six studies.[4,6,22,23,25,36] Elevated serum potassium is reported as an indicator in seven studies.[6,21,23,24,37–39] Serum creatinine level above 3 mg/dl is also shown to correlate with death.[4,24] Acidosis, which may indicate pulmonary or renal involvement, is reported as a predictor of death in two studies.[7,40] A statistically significant association between established renal failure and death is reported in another two studies.[8,41]

Bacterial infection, insults from the inflammatory process, and hemodynamic disturbances are thought to contribute to renal failure in leptospirosis. The basic pathology is an interstitial nephritis with tubular necrosis resulting in ARF. Manifestations of renal involvement vary from laboratory evidence (changes in urinary sediment, evidence of tubular dysfunction) to clinically defined ARF with hyperkalaemia and elevated creatinine.[42] It is estimated that 44–67% of the patients can have renal failure in leptospirosis.[6] The mortality rates published from such involvement vary according to the locality studied (26% in Sri Lanka, 36% in Barbados).[43,44]

Central nervous system involvement is another predictor of severe disease and death. Covic *et al*.[45] published a retrospective study on 51 patients with severe leptospirosis. Altered level of consciousness was seen in all patients who died, but this was only 33% in the patients who survived (*P* < 0.05). Esen *et al*.[39] also reported a significantly high rate of altered mental status (*P* = 0.01) in deceased patients compared to survivors (retrospective analysis of 72 patients). According to Ko *et al*.,[8] who studied 326 patients prospectively, altered mental status was the strongest predictor of death (OR, 9.12). Mathew *et al*.,[27] reporting on a retrospective and a prospective study of 31 patients with neuroleptospirosis, suggested that deep altered sensorium at presentation correlated with mortality (*P* < 0.037). Meningism and disorientation (OR, 5 and 10.6, respectively) are also reported as mortality indicators by Pappachan *et al*.[21]

Three studies have independently associated altered mental status with death. However, many factors can cause altered mental status in severe leptospirosis, and thus this clinical feature is lacking in specificity. These studies also do not specify the criteria used to assess altered mental status and hence it is difficult to assess the uniformity of the findings.

Cardiac involvement in leptospirosis is underestimated. Describing the findings of 44 autopsies with a presumptive cause of death with leptospirosis-related complications, Charkurkar *et al*.[46] reported interstitial myocarditis in 100% of the cases, with involvement of the epicardium or endocardium (39%), valves (36%), coronary arteries (51%) and aorta (56%). However, clinical manifestations of cardiac involvement were limited. Dupont *et al*.,[25] in his retrospective study, reported several ECG abnormalities, including, sinus tachycardia and bradycardia, repolarization abnormalities, conduction abnormalities, atrial/ventricular extrasystoles and atrial fibrillation. Only repolarization abnormalities were shown to be a predictor of mortality.

Evidence for hepatic dysfunction is prominent during the clinical course of leptospirosis. However, many of these are not reported as predictors of mortality. Pappachan *et al*.[21] reported that serum bilirubin above 15 mg/dl is an indicator of death (OR, 5.4) while Cetin *et al*.[6] reported a much higher limit of 36 mg/dl. Similarly, Chang *et al*. reported that a high Aspartate transaminase (AST)/alanine transaminase (ALT) ratio in late disease is associated with death.[28] Bleeding diathesis was reported as a mortality indicator by Covic *et al*.[45]

MODS, which is the involvement of more than one organ system, has been shown to be associated with death in four studies [Table 4].[7,30,40,45]

## PROBLEMS IN CLASSIFICATION AND POTENTIAL FOR FURTHER DEVELOPMENT

There were a few other predictors of mortality that could not be classified under the PIRO model. These were mainly treatment related. Andrade *et al*.,[47] in a two-sample cohort study, have shown that prompt and daily dialysis had a survival advantage over alternate-day dialysis for patients in ARF (*P* = 0.01). In their study involving 35 critically ill patients with ARF, Vieira *et al*.[40] stated that length of stay in ICU is positively associated with mortality. A review in Thailand has shown that blood exchange therapies (hemodialysis, hemofiltration) were associated with lower mortality (0 vs. 10%), shorter recovery time (8 vs. 16 days) and faster reduction in serum levels of bilirubin, urea and creatinine when compared to peritoneal dialysis.[48]

There is a place for further research to determine the role of gender, infecting serovars, clinical signs, inflammatory markers, cytokines, biochemical parameters and markers of hepatic dysfunction as prognostic indicators. It is interesting to note that several recent studies in India have shown that clinical manifestations like pancreatitis and syndrome of inappropriate secretion of anti-diuretic hormone were more common than previously thought. In one study, the majority of patients with these presentations were anicteric. There may be many other predictors of mortality yet unidentified in relation to these presentations.[49]

## LIMITATIONS

Only articles published in English were included in the review. Therefore, it is possible that certain important papers were missed. Similarly, relevant information published beyond the time limits of our search have not been taken into account. Such gaps in data may affect the validity and generalizability of the conclusions.

## CONCLUSIONS

This review was synthesized as a concept paper to direct further research into mortality indicators of leptospirosis.

It attempts to

- summarize and discuss evidence on predictors of mortality in severe letospirosis
- develop a classification system for such factors based on the PIRO concept used for staging of severe sepsis

On analyzing the evidence on mortality indicators, some predictors (age, renal/pulmonary involvement and multiple organ dysfunction) have been repeatedly and independently confirmed by different authors. Still, some other associations such as the impact of inflammatory markers and leukocytosis have not been assessed in detail. Many identified risk factors could be categorized into the four domains of the PIRO concept. However, treatment-related factors could not be incorporated into the existing model. In this regard, we propose to add a fifth domain of treatment (T) when modifying the PIRO concept for leptospirosis.

While we managed to classify the mortality indicators, a scoring system to quantify the relative risk could not be developed due to differences of the methodologies and non-comparability of data. Such a scoring/staging system would be more scientifically valid if developed by a prospective study specially designed for such a purpose. It is hoped that our work will be a starting point to the development of a staging system to predict the severity of leptospirosis.[50]
